# Use of an application on the measles vaccine for Warao indigenous refugees in Brazil

**DOI:** 10.1590/0034-7167-2023-0253

**Published:** 2024-03-11

**Authors:** Bárbara Lopes Paiva, Ingrid Bentes Lima, Laura Maria Vidal Nogueira, Ivaneide Leal Ataíde Rodrigues, Samantha Pereira Caldas, Marizete Lopes Andrade, Arthur da Silva Costa Pedroza, Anderson Raiol Rodrigues

**Affiliations:** I Universidade Federal do Pará. Belém, Pará, Brazil; II Universidade do Estado do Pará. Belém, Pará, Brazil

**Keywords:** Indigenous Peoples, Immunization, Measles, Refugees, Mobile Health, Pueblos Indígenas, Inmunización, Sarampión, Refugiados, Mobile Health, Povos Indígenas, Imunização, Sarampo, Refugiados, Celular de Saúde

## Abstract

**Objective:**

To evaluate the need to develop an application with information about the measles vaccine for Warao indigenous people.

**Methods:**

This was a quantitative study conducted at the Espaço de Acolhimento Tapanã refugee shelter in the city of Belém, Pará, Brazil. The study sample was selected for convenience. Data were analyzed descriptively using Bioestat 5.0 software.

**Results:**

Twenty-one Warao indigenous individuals were interviewed. It was identified that 91% (n=20) had lost their vaccination card; 91% (n=20) stated they had lost their vaccination card more than three times, and 91% expressed interest in an application to store their vaccination information.

**Conclusions:**

The research provided important information for the development of a health application named WaraoMedI (Warao Measles Diversity Indigenous), as well as offered nursing professionals evidence about the challenges Warao indigenous refugees face in self-managing their vaccination information.

## RESUMO

**Objetivo:**

avaliar a necessidade de desenvolver um aplicativo com informações sobre a vacina antissarampo para indígenas Warao.

**Métodos: Estudo quantitativo, realizado no abrigo de refugiados chamado Espaço de Acolhimento Tapanã, na cidade de Belém, Pará, Brasil. Amostra do estudo foi por conveniência.:**

Os dados foram analisados de forma descritiva, por meio do software Bioestat 5.0.

**Resultados:**

foram entrevistados 21 indígenas Warao. Identificou-se que 91% (n=20) perderam o cartão de vacina; 91% (n=20) afirmaram que perderam o cartão de vacina mais de 3 vezes e 91% afirmaram que gostariam de um aplicativo para guardar suas informações de vacina.

**Conclusões:**

A pesquisa ofereceu informações importantes para a construção de um aplicativo em saúde chamado WaraoMedI (Warao Mesles Diversity Indigenous), assim como disponibilizou aos profissionais de enfermagem evidências sobre as dificuldades de os indígenas Warao refugiados autogerenciarem informações sobre vacina.

## RESUMEN

**Objetivo:**

evaluar la necesidad de desarrollar una aplicación con información sobre la vacuna contra el sarampión para los indígenas Warao.

**Métodos:**

Estudio cuantitativo, realizado en el refugio de refugiados llamado Espacio de Acolhida Tapanã, en la ciudad de Belém, Pará, Brasil. La muestra del estudio fue por conveniencia. Los datos fueron analizados de forma descriptiva, mediante el software Bioestat 5.0.

**Resultados:**

se entrevistaron a 21 indígenas Warao. Se identificó que el 91% (n=20) perdieron su tarjeta de vacunación; el 91% (n=20) afirmó que perdió la tarjeta de vacunación más de 3 veces y el 91% afirmó que les gustaría una aplicación para guardar su información de vacunación.

**Conclusiones:**

La investigación proporcionó información importante para la construcción de una aplicación de salud llamada WaraoMedI (Warao Measles Diversity Indigenous), así como proporcionó a los profesionales de enfermería evidencia sobre las dificultades de los indígenas Warao refugiados para autogestionar información sobre vacunación

## INTRODUCTION

We are currently facing a significant global health issue known as the refugee crisis. In 2020, 893 million people were forcibly dis-placed worldwide. By 2021, this number had surprisingly increased to 100 million, exceeding 1% of the total global population^(1, 2)^. Never in history have such high levels of population displacement been recorded on the planet^(2)^. In this scenario, a group of Warao indigenous refugees has drawn the attention of international public health authorities and, in Brazil, especially in Pará, in the city of Belém, due to the high migratory flow of this ethnic group^(2, 3)^.

In 2020, the UN Refugee Agency (UNHCR) registered over 4,281 Warao indigenous people in Brazil since 2017. In June 2020, it was estimated that 1,000 of them were living in various municipalities of the state of Pará. That same year, an increase in arrivals was observed in Belém, Santarém, Ananindeua, Itaituba, Altamira, Redenção, Marabá, Parauapebas, and Óbidos. The city of Belém stands out with an approximate number of 450 Warao indigenous people, representing 20% of this ethnic group present in the entire state of Pará^(3)^.

The Warao indigenous people exhibit a migration profile of mass individuals with low vaccination coverage^(4, 5)^. They possess unique cultural traits, such as begging and limited access to health services in their country of origin^(6, 7)^.

This situation could lead to the introduction or reintroduc-tion of diseases, especially if the displaced populations live in temporary accommodations with poor sanitation practices and water storage, in addition to limited access to healthcare^(8)^, as has been the reality for many Warao indigenous refugees crossing the Brazilian border^(3)^.

In 2017, the first records of the Warao indigenous people set-tling in the city of Belém were documented. The following year, in 2018, a measles outbreak occurred, with five children of this ethnicity diagnosed with the disease^(9)^.

In the global immunization strategy called the Immunization Agenda 2030 (IA2030), launched in 2020, an alert was raised about the need for innovative solutions for vaccinating vulnerable popula-tions, in order to advance the achievement of the United Nations Sustainable Development Goal 3: health and well-being. Furthermore, IA2030 envisions a world where everyone, of all ages, everywhere, benefits from vaccines to improve health and well-being^(10, 11)^.

## OBJECTIVE

To assess the necessity of developing an application with information about the measles vaccine for the Warao indigenous people.

## METHODS

### Ethical Aspects

To conduct this study, authorization was required from the Belém Municipal Health Department (SESMA) and the João Paulo II Foundation (FUNPAPA), which is responsible for managing Social Assistance Policy in the municipality of Belém. Permission was also needed to access the services of the Street Clinic (CnaR), affiliated with SESMA, and the Tapanã Reception Space (EA), managed by FUNPAPA.

The research project was presented to the community of Warao indigenous refugees at EA – Tapanã on February 4, 2021, in accordance with CNS Resolution No. 304/2000, Sections III.2.4 and IV.1. The Letter of Consent was drafted and signed in Por-tuguese, Spanish, and Warao. As most indigenous refugees at the site are learning Portuguese, it was crucial to communicate the information to the community and retain document copies in a native language. After obtaining the consents, the project was submitted to the Ethics Committee (CEP) and the Brazilian National Health Council (CNS) for approval.

Online, informed consent was obtained from all study participants.

### Study Design, Period, and Location

**Figure 1:**
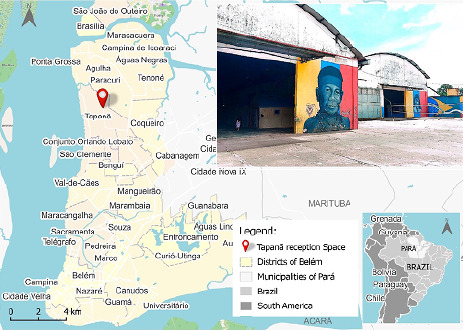
Research location, Belém, Pará, Brazil, 2023

This research utilized a quantitative approach, guided by the STROBE framework of the EQUATOR Network. Data were gathered through interviews conducted from March 16 to April 17, 2023, with Warao indigenous people residing at the EA–Tapanã, the sole official SESMA facility designated for the development of support and protection activities for Warao indigenous people in refugee situations in Belém, Pará, Brazil^(12)^ (Figure 1).

This article is a part of a doctoral research project aimed at developing an application named WaraoMedI (Warao Measles Diversity Indigenous). The Human-Centered Design (HCD) or Design Thinking method was selected. This approach involves an empathetic design process with rapid and iterative prototyping in healthcare^(13)^. The method includes three phases: Listen, Create, and Deliver. In the ‘Listen’ phase, the Warao indigenous people’s needs regarding cellphone usage and vaccination information management were identified. In the ‘Create’ phase, brainstorm-ing techniques, visual layout, and synthesis of need statements were used to guide rapid prototyping and collect feedback from end-users. The final ‘Deliver’ phase utilized the System Usability Scale (SUS), a Likert scale, for preliminary validation of the app and to evaluate its effectiveness, efficiency, and user satisfac-tion. This article focuses only on the results of the ‘Listen’ phase. It is important to note that the ‘Create’ and ‘Deliver’ phases were completed and met the Warao users’ needs, but their results will be discussed in future scientific publications.

### Population and Selection Criteria

The study participants were selected for convenience. The research involved 21 Warao indigenous individuals who frequented the EA-Tapanã as refugees or refugee status applicants. The partici-pants were of both genders, including children and adolescents, provided they had a family member authorize their participation and voluntarily agreed to participate. The criteria included those who spoke Portuguese, Spanish, or Warao, and individuals free from addictions, subordination, or intimidation, in accordance with CNS Resolution 466/2012, items II-23 and 24. Excluded were individuals with impaired vision, intellectual disabilities, drug involvement, mental illness, those in highly vulnerable situations due to recent physical and sexual abuse, the sick, and those who worked or studied during morning and afternoon hours.

### Data Collection Instrument

Initially, studies by Paradis et al. (2018)^(14)^ and Louka et al. (2019)^(15)^, which investigated technology use by refugees and vaccination itineraries, were reviewed to create a script tailored to the Warao’s reality and support the interviews. Following this, the first version of the script was developed, divided into five sections: A - Socio-economic data; B - Cellphone usage by the Warao indigenous person; C - Cellphone usage by the family of the Warao indigenous person; D - Information about vaccines and knowledge of measles; and E - Final recommendations from participants regarding additions to the app. The script was pre-tested from May 8 to 17, 2020, with 10 Warao indigenous people served by the CnaR who did not reside at the EA – Tapanã. This pre-test refined the data collection form, confirmed the need for an interpreter for the interviews, and assessed the time required for each interview, thus estimating the average duration for the research. The Warao who participated in the pre-testing did not interact with the study population.

### Study Protocol

Using a spreadsheet, in collaboration with the EA – Tapanã professionals, the Warao indigenous people who met the study’s inclusion criteria and were willing to participate in online interviews were identified. The option for online interviews was provided, as the EA – Tapanã is equipped with a computer room. The availability of both online and in-person interviews was intended to foster a connection with the audience, encouraging active participation and adherence to scheduled interviews. Of the 100 indigenous people living at the EA – Tapanã, 60 were eligible for the study, and 21 consented to participate, with only two choosing in-person interviews. The interviews were scheduled in advance via Google Meet videoconferencing, including the in-person interviews, and typically lasted 30 to 45 minutes. They were recorded on a voice recorder, with prior authorization from the participants. Before the interviews, the Informed Consent Form (ICF) was presented to adults and the guardians of children and/or adolescents, and the Informed Assent Form (IAF) was presented to children and/or adolescents, with digital signatures obtained from the participants online.

### Data Analysis

The data were organized in electronic spreadsheets and ana-lyzed using descriptive statistics (mean and standard deviation). The corrected chi-square test and Pearson’s chi-square test were applied using Bioestat 5.0 software.

## RESULTS

### Socioeconomic Characterization of the Warao Indigenous People

The study involved 21 Warao indigenous individuals residing at the EA-Tapanã, all (100%, N=21) originating from Venezuela, specifically from the Delta Orinoco region. Regarding gender, 52.38% (n=11) were female and 47.62% (n=10) male. The age distribution was as follows: 8-17 years, 19% (n=4); 18-27 years, 14% (n=2); 28-37 years, 29%; 38-47 years, 29% (n=4); 48-57 years, 10% (n=3); and 58-67 years, 10% (n=2) (Table 1). In terms of education, 9.52% (n=2) had no formal education; 9.52% (n=2) had incom-plete primary education; 33.33% (n=7) had completed primary education; 33.33% (n=7) had incomplete secondary education; and 14.29% (n=3) had completed secondary education (Table 1).

**Table 1:** Socioeconomic Data of the Warao Indigenous Refugees, Belém, Pará, Brazil, 2023

Variable	N= (21)	%	*p* value
Gender	µ = 10.50	σ = 0.71	
Female	11	52	0.827ns
Male	10	47	
Age Range (in years)	µ = 3.50	σ = 1.52	
8-17	4	19	0.096ns
18-27	2	10	
28-37	6	29	
38-47	4	19	
48-57	3	14	
58-67	2	10	
Country of Origin	-	-	
Venezuela	21	100	-
Region of Origin	-	-	
Delta Orinoco	21	100	
Language	-	-	
Portuguese	21	100	-
Warao	21	100	
Spanish	21	100	
Work and Income	-	-	
Government aid only	18	85.70	0.000**
Formal employment	1	4.70	
Informal employment	1	4.70	
Employment and government aid	1	4.70	
Year of Arrival in Belém	µ = 4.75	σ = 3.77	
2018	4	19	0.029*
2019	4	19	
2021	1	4.70	
2022	10	47.60	
Did not respond	2	9.50	
Level of Education	µ = 4.20	σ = 2.59	
Completed Elementary Education (1st degree)	7	33.30	0.172ns
Incomplete Elementary Education (1st degree)	2	9.50	
Incomplete High School (2nd degree)	7	33.30	
Completed High School	3	14.30	
No Education	2	9.50	

Notes: * Corrected chi-square test; ** Pearson chi-square test; * µ represents the mean; *σ symbolizes the standard deviation; *σ symbolizes the standard deviation.

Regarding employment and receipt of government aid, 85.71% (n=8) received aid; 4.76% (n=1) were employed in formal or self-employment; and 4.76% (n=1) combined work with receipt of aid. Two women worked as artisans, and one man as a seller of internet services for a mobile telephone operator (Table 1). The values in Table 1 were statistically significant, with a p value ≤0.05, indicating a normal distribution of data, as evidenced by the µ and σ values of the variables.

### Identification of Mobile Digital Technology Among the Warao Indigenous People

All participants (100%, N=21) owned cellphones, with 86% (n=16) having Samsung models and 14% (n=5) Motorola models. Regarding app usage, all (100%, N=21) used Facebook; 86% (n=16) used Instagram and YouTube; and 62% (n=13) used Telegram. In terms of internet usage, 95% (n=20) stated they used it continuously, primarily for communication with family in Venezuela and for accessing vari-ous types of information. Despite financial limitations, it was noted that respondents prioritized maintaining at least one cellphone per family group.

### Mobile Digital Technology in the Families of the Warao Indigenous People

All respondents (100%, N=21) reported that other family members also owned cellphones, with 54% (n=12) being spouses, 27% (n=6) children, and 19% (n=4) parents. The majority (76%, n=16) used Motorola phones, and 24% (n=5) used Samsung. Regarding social media usage, 45% (n=9) used Facebook, 17% (n=3) Instagram, 19% (n=5) WhatsApp, 17% (n=3) YouTube, and 2% (n=1) TikTok.

### Information about Vaccination and Knowledge of Measles

As shown in Table 2, 91% (n=20) were vacci-nated upon arrival in Brazil, 73% (n=16) received vaccinations more than three times, and 64% (n=14) were vaccinated against measles. The same percentage (91%, n=20) reported losing their vaccination card more than three times. Regarding symptoms of measles, 91% (n=20) recognized red spots on the skin; 82% (n=18) experienced a sore throat; 91% (n=20) had a high fever; 82% (n=18) had redness in the eyes; and 23% (n=5) had white spots inside the mouth.

**Table 2:** Information on Immunization Records and Measles Vaccination, Belém, Pará, Brazil, 2023

Variable	N=21	%	*p* value^(1)^
When you arrived in Brazil, were you vaccinated?	µ = 10.50	σ = 13.44	
Yes	20	95	0.000**
No	1	5	
Don't remember	0	0	
How many times have you been vaccinated in Brazil?	µ = 7.00	σ = 7.81	
More than 3 times	16	73	0.000**
More than 5 times	2	9	
Don't remember	3	14	
Do you remember if you were vaccinated against Measles?	µ = 7.00	σ = 6.24	
Yes	14	64	0.000**
No	5	23	
Don't remember	2	9	
Did you lose your vaccination card?	µ = 10.50	σ = 13.44	
Yes	20	91	0.000**
No	1	5	
How many times have you lost your vaccination card?	µ = 10.50	σ = 13.44	
More than 3 times	20	91	0.000**
More than 5 times	1	5	
What are the symptoms of Measles for you?	µ = 16.20	σ = 6.34	
Red spots on the skin that do not itch	20	91	0.042**
Sore throat	18	82	
High fever	20	91	
Redness in the eyes	18	82	
White spots inside the mouth	5	23	

Note: * Corrected chi-square test; ** Pearson chi-square test; * µ represents the mean; *σ symbolizes the standard deviation.

### Warao Indigenous Preferences for Health Apps

The most common responses from the Warao indigenous people indicated that 91% (n=20) would like to have a mobile app to store photos of their vaccination cards, considering it important to receive guidance on the signs and symptoms of measles and information on how to seek help in case of illness. They also emphasized that an app would be well received by the Warao community, provided it is available in Warao, Spanish, and Portuguese languages.

## DISCUSSION

This study provides insights into how an application containing information about the measles vaccine should be developed to best meet the unique needs of the Warao indigenous refugees. Although a high percentage (100%) of participants owned smartphones, none of the respondents were active users of health applications. When asked if they would use an app to learn about the measles vaccine, 91% of participants expressed interest in using such a tool, especially if it were translated into Spanish and Warao.

The high acceptability for the development of the application is similar to that in other studies^(16, 17)^. In the study by Paradis et al. (2018) ^(14)^, the acceptability of refugees using an app to manage their vac-cines was analyzed, where 76% indicated they would use a version translated into their primary language. The VivI Health Survey app, discussed in the study by Stopa et al. (2020)^(18)^, also identified interest among young refugees in Germany. A literature review by Mancini et al. (2019)^(19)^ highlighted the importance of mobile technologies in the daily lives of refugees for various purposes.

Typically, the Warao indigenous refugees face dangerous cross-ings and often lose important documents, such as vaccination cards, as identified in the study by Filler et al. (2020)^(20)^. In addition to the challenges of displacement, refugees must adapt to vaccination policies in host countries^(21)^, facing linguistic barriers and a lack of humanization, as identified in various studies^(22)^. In contrast, a national survey of Bolsa Família beneficiaries showed a high rate of vaccination registration in children^(23)^, highlighting differences in vaccination management between adults and children.

Refugees, especially those fleeing wars, persecution, or natural disasters, come from regions with weak or disrupted health systems and face unique challenges in accessing healthcare. Many of them, due to a lack of health information or education in their countries of origin, are unaware of disease definitions or symptoms, or have only vague knowledge about them^(24)^. The Warao, in addition to the chal-lenges faced, have ethnic and cultural characteristics that demand specific health technologies. Notable practices include culturally oriented food begging, considered by them as a form of work, and traditional approaches to combating diseases and healing practices.

Furthermore, they face social stigmas, child mortality, xeno-phobia, and a high death rate related to the HIV virus^(6)^. A study with Rohingya refugees in Bangladesh showed that 63.1% of parents had good practices in immunizing their children, related to determinants of vaccine coverage such as education, family income, father’s occupation, and experiences of child loss^(25)^.

A systematic review by Ekezie et al. (2022)^(26)^ highlighted critical factors associated with the vaccination of disadvantaged groups in Europe, revealing that the recall of previous vaccines received was poor and that the perception of risk, the severity of the disease, and the benefits of vaccination varied according to the level of education and language.

### Study Limitations

This study has limitations. The sample, relatively small and specific, limits the generalization of the results to other refugee contexts. The lack of diversity in the sample may not fully reflect the experiences and needs of the Warao indigenous refugees. Additionally, the ab-sence of an interpreter to mediate all dialogues, considering some participants speak Spanish and Warao, made it difficult to understand the accounts of the vaccination journey in a refugee situation.

### Contributions to Nursing

The study results contribute to the development of strategies aimed at improving measles vaccination coverage, particularly in the field of indigenous health. The findings assist in nursing practice, allowing professionals to use technological resources in health for planning and executing immunization-related activities. The identification of the need for an application with measles vaccination information for the Warao indigenous people contributed to the development of the WaraoMedI (Warao Measles Diversity Indigenous) application, making the process of secondary health prevention more dynamic, practi-cal, collaborative, and effective.

## CONCLUSION

We demonstrated the need for Warao indigenous refugees to seek information through the use of cell phones. Thus, the interest of this group in the development of the WaraoMedI (Warao Measles Diversity Indigenous) application was identified, aiming to store vac-cination data and prevent the loss of records, thereby avoiding the need to repeat vaccinations. The findings also showed the Warao’s interest in self-managing their vaccination information and actively participating in the development of health-related applications.
